# Late gastrointestinal tissue effects after hypofractionated radiation therapy of the pancreas

**DOI:** 10.1186/s13014-015-0489-2

**Published:** 2015-09-04

**Authors:** Adnan Elhammali, Mukund Patel, Benjamin Weinberg, Vivek Verma, Jingxia Liu, Jeffrey R. Olsen, Hiram A. Gay

**Affiliations:** Department of Radiation Oncology, Washington University School of Medicine, 4921 Parkview Place, Campus Box 8224, St. Louis, MO 63110-63110 USA; Department of Radiation Oncology, The Brody School of Medicine at East Carolina University, Greenville, NC USA; Department of Radiation Oncology, Wayne State University, Detroit, MI USA; Department of Radiation Oncology, University of Nebraska Medical Center, Omaha, NE USA; Division of Biostatistics, Washington University School of Medicine, St. Louis, MO USA

## Abstract

**Background:**

To consolidate literature reports of serious late gastrointestinal toxicities after hypofractionated radiation treatment of pancreatic cancer and attempt to derive normal tissue complication probability (NTCP) parameters using the Lyman-Kutcher-Burman model.

**Methods:**

Published reports of late grade 3 or greater gastrointestinal toxicity after hypofractionated treatment of pancreatic cancer were reviewed. The biologically equivalent dose in 1.8 Gy fractions was calculated using the EQD model. NTCP parameters were calculated using the LKB model assuming 1–5 % of the normal tissue volume was exposed to the prescription dose with α/β ratios of 3 or 4.

**Results:**

A total of 16 human studies were examined encompassing a total of 1160 patients. Toxicities consisted of ulcers, hemorrhages, obstructions, strictures, and perforations. Non-hemorrhagic and non-perforated ulcers occurred at a rate of 9.1 % and were the most commonly reported toxicity. Derived NTCP parameter ranges were as follows: n = 0.38–0.63, m = 0.48–0.49, and TD_50_ = 35–95 Gy. Regression analysis showed that among various study characteristics, dose was the only significant predictor of toxicity.

**Conclusions:**

Published gastrointestinal toxicity reports after hypofractionated radiotherapy for pancreatic cancer were compiled. Median dose was predictive of late grade ≥ 3 gastrointestinal toxicity. Preliminary NTCP parameters were derived for multiple volume constraints.

## Background

With an overall 5-year survival of 5 % and a 5-year survival of 20 % after surgical resection, pancreatic cancer has an extremely poor prognosis [1]. It is the 9^th^ most common malignancy in the United States, but the 5^th^ most common cause of cancer-related death [2]. The aggressive nature of this cancer is partly due to its late presentation and the intimate anatomic relationship between the pancreas and adjacent structures, namely the duodenum, stomach, liver, bile ducts, spleen, and the great vessels and their branches. Whether surgical, pharmacological, or radiological, any pancreatic treatment must attempt to preserve the integrity and function of these structures. The only potentially curative treatment for pancreatic cancer is surgical, although chemotherapy or chemoradiotherapy are often employed in the adjuvant setting [3, 4]. Radiation therapy is also used in the setting of unresectable disease for local control and symptomatic palliation of pain and obstruction.

Conventionally fractionated treatments are lengthy, may delay needed systemic therapy, and have not been shown to be curative in unresectable disease. For these reasons, hypofractionated schemes have sometimes been employed, including stereotactic radiosurgery (SRS), stereotactic body radiotherapy (SBRT), and intraoperative radiotherapy (IORT).

Because of its close anatomic association with the pancreas and its relative radiosensitivity, the small bowel and stomach are the major dose-limiting organs in radiation treatment of the intact pancreas. Because of the poor long-term survival of this patient population, acute side effects in the gastrointestinal tract are better characterized than late effects. The objective of this work was to compile literature reports of grade 3 or greater late toxicities in hypofractionated radiation treatment of pancreatic cancer and attempt to derive normal tissue complication probability (NTCP) parameters using the Lyman-Kutcher-Burman (LKB) model [5].

## Methods

### Review criteria

A series of PubMed searches were performed looking for English-language original articles that reported gastrointestinal toxicity in humans following treatment of pancreatic cancer with external beam radiation therapy. Over 200 papers fit our general search criteria, and these were carefully screened for papers that reported serious (grade 3 or above) late gastrointestinal complications from treatment with hypofractionated radiotherapy, without regard to technique. An attempt was made to select papers with specific mention of duodenal toxicity, but we also included papers reporting late effects in the stomach, small intestine, and other gastrointestinal organs. We also made an effort to avoid studies of patients whose complications were reported in previous publications. For the purposes of this review, late complications were considered to be those that occurred after a minimum of 3 months of follow-up. The eligible articles were published from 1981 to 2013.

### Equivalent dose calculation and nomenclature

The biologically equivalent dose in *f* Gy fractions for a total dose *D* Gy given in *d* Gy fractions using an r α/β ratio is defined as:

$$ EQ{D}_r^f=D\left(\frac{d+r}{f+r}\right) $$

For example, 60*Gy*_3_^2^ means a biologically equivalent dose of 60 Gy in 2 Gy fractions using an α/β ratio of 3. To simplify the comparison of different hypofractionated schedules, we will use biologically equivalent doses defined in standard 1.8 Gy fractions, i.e.:

$$ EQ{D}_r^{1.8}=eG{y}_r^{1.8}=D\left(\frac{d+r}{1.8+r}\right) $$

### NTCP LKB Model, Maximum likelihood fitting, and confidence intervals

Normal tissue complication probity (NTCP) were calculated using the Lyman-Kutcher-Burman (LKB) model as follows [5]:

$$ \mathrm{NTCP}=\frac{1}{\sqrt{2\pi }}\underset{-\infty }{\overset{t}{\int }}e-\frac{x^2}{2}\mathrm{d}x $$

where *t* is defined as

$$ t=\frac{D_{eff}-T{D}_{50}}{mT{D}_{50}} $$

, and *D*_*eff*_ is

$$ {D}_{eff}={\left({\displaystyle {\sum}_i{v}_i{D}_i^{\raisebox{1ex}{$1$}\!\left/ \!\raisebox{-1ex}{$n$}\right.}}\right)}^n $$

and represents the dose that if distributed evenly across the volume, produces the same complication probability as the actual dose distribution represented by the summation. Variables *v*_*i*_ and *D*_*i*_ are the volume and dose of each bin of the dose volume histogram (DVH). Because the original DVH data was unavailable, hypothetical DVHs were constructed assuming 1–5 % of the duodenum received the study’s prescribed dose while the remaining volume received no dose. TD_50_ is the dose that produces a 50 % complication probability if delivered uniformly to the organ. The variable *m* relates to the slope of the integral of the normal distribution and *n* denotes if the tissue is parallel or serial. Optimal solutions were obtained using the maximum likelihood method by maximizing the following function:

$$ \ln L={\displaystyle {\sum}_i\left\{\left({n}_i-{q}_i\right) \ln \left(1-NTC{P}_i\right)+{q}_i \ln \left({\mathrm{NTCP}}_i\right)\right\}}, $$

such that *n*_*i*_ represents the total number of patients and *q*_*i*_ the number of patients that developed complications within bin *i* of radiation dose. The profile likelihood method was then used to calculate 95 % confidence intervals for *TD*_*50*_, *m*, and *n* [6].

### Statistical analysis

Linear regression model was used in univariate analysis of toxicity rate. Variables of interest included median follow-up, radiation dose level, number of dose fractions, % of patients receiving chemotherapy, % of patients receiving surgery, and median overall survival. All statistical tests were two-sided using an α = 0.05 level of significance. SAS version 9.3 (Cary, NC) was used to perform the above statistical analysis.

## Results

### Study characteristics

A total of 16 human studies and two canine studies were examined and are summarized in Tables 1 and 2 [7–24]. Two studies included characteristic and outcome data for two separate cohorts and were therefore analyzed independently [19, 21]. The total number of patients treated was 1160 with a median of 60 patients per study (range: 19–210). The median length of follow-up in months among studies that reported a follow-up time was 11.6 (range 3–28). The median EQD_4_^1.8^ was 125 Gy (range: 50–209).

**Table 1:** Human GI toxicity rates from studies using hypofractionated radiotherapy for pancreatic cancer

Author (reference)	N	Median follow-up (months)	Radiotherapy	*EQD*_4_^1.8^	Chemo (%)	Surgery (%)	Late GI complications	Complication rate	Total GI toxicity rate
Mahadevan et al. [7]	36	24	30 Gy 3 fx^a^ (median)	72	86.1 %	None	GI bleeding requiring transfusion, grade ≥ 3	2/36 (5.6 %)	5.6 %
Mahadevan et al. [8]	39	21	24 Gy 3 fx^a^ (median)	50	100 %	None	GI bleeding requiring transfusion, grade 3	2/39 (5.1 %)	7.7 %
							Gastric outlet obstruction, grade 3	1/39 (2.6 %)	
Lominska et al. [9]	28	5.9	21 Gy 3 fx^a^ + 50.4 Gy^c^ (median)	90	71 %	29 %	Bowel obstruction, grade 3	1/28 (3.6 %)	7.1 %
							Gastric perforation, grade 3	1/28 (3.6 %)	
Chang et al. [10]	77	6	25 Gy 1 fx^a^ (exact)	125	96 %	None	Biliary stricture, grade 3	2/77 (2.6 %)	9.1 %
							Duodenal stricture, grade 3	1/77 (1.3 %)	
							Small bowel perforation, grade 4	1/77 (1.3 %)	
							Gastric ulcer, grade 3	3/77 (3.9 %)	
Hoyer et al. [11]	22	3	45 Gy 3 fx^a^ (median)	147	None	13.6 %	Duodenum or stomach severe mucositis^d^	2/22 (9.1 %)	22.7 %
							Duodenum or stomach ulceration^d^	2/22 (9.1 %)	
							Stomach perforation^d^	1/22 (4.5 %)	
Chuong et al. [12]	73	10.5	30 Gy 5 fx^a^ (median)	52	100 %	56 %	GI bleeding requiring embolization, grade 3	3/73 (4.1 %)	5.5 %
							Anorexia resulting in feeding tube placement, grade 3	1/73 (1.4 %)	
Schellenberg et al. [13]	20	11.8	25 Gy 1 fx^a^ (exact)	125	100 %	None	Duodenal perforation, grade ≥ 3	1/20 (5.0 %)	5.0 %
Didolkar et al. [14]	85	Unknown	25.5 Gy 3 fx^a^ (median)	55	100.0 %	16.5 %	Late duodenitis (upper GI hemorrhage or obstruction), grade ≥ 3	7/85 (8.2 %)	8.2 %
Polistina et al. [15]	23	9	30 Gy 3 fx^a^ (exact)	72	100 %	None	Late GI toxicity, grade ≥ 3	0/23 (0 %)	0.0 %
Rwigema et al. [16]	71	12.7	24 Gy 1 fx^a^ (median)	116	90 %	39 %	Late GI toxicity, grade ≥ 3	0/71 (0 %)	0.0 %
Ogawa et al. [17]	210	26.3	25 Gy 1 fx^b^ (median)	125	54.3 %	100 %	GI toxicity unspecified, grade 3	3/210 (1.4 %)	3.3 %
							Colitis, grade 4	1/210 (0.5 %)	
							GI bleeding, grade 4	2/210 (1.0 %)	
							Ileus, grade 4	1/210 (0.5 %)	
Willett et al. [18]	150	17	20 Gy 1 fx^b^ + 50.4 Gy^c^ (median)	133	100 %	82 %	Bleeding secondary to duodenal ulcer or erosion requiring medical intervention^d^	16/150 (10.6 %)	15.0 %
							Fatal duodenal bleeding, Grade 5	2/150 (1.3 %)	
							Duodenal obstruction^d^	1/150 (0.6 %)	
							Abdominal wall dehiscence^d^	1/150 (0.6 %)	
							Other^d^	2/150 (1.3 %)	
Mohiuddin et al. [19]	49	28	20 Gy 1 fx^b^ + 50 Gy^c^ (median)	133	100 %	None	Cholangitis, grade ≥ 3	2/49 (4.1 %)	16.3 %
							GI bleeding, gastric antrum or transverse colon, grade ≥ 3	3/49 (6.1 %)	
							Bowel obstruction, grade ≥ 3	2/49 (4.1 %)	
							Enteritis, grade ≥ 3	1/49 (2 %)	
Nishimura et al. [20]	55	Unknown	26 Gy 1 fx^b^ + 44 Gy^c^ (mean)	178	34.2 %	100 %	GI ulcer (non-perforating)^d^	11/55 (20.0 %)	32.7 %
							Intestinal perforation^d^	2/55 (3.6 %)	
							Abdominal abscess^d^	3/55 (5.5 %)	
							Ileus^d^	2/55 (3.6 %)	
	71		29.3 Gy 1 fx^b^ + 41 Gy^c^ (mean)	209	27.4 %	None	GI ulcer (non-perforating)^d^	7/71 (9.9 %)	19.7 %
							Intestinal perforation^d^	2/71 (2.8 %)	
							Abdominal abscess^d^	1/71 (1.4 %)	
							Duodenal fibrosis^d^	3/71 (4.2 %)	
							Ileus^d^	1/71 (1.4 %)	
Okamoto et al. [21]	68	Unknown	20 Gy 1 fx^b^ + 50 Gy^c^ (median)	135	8.8 %	94.1 %	Duodenal ulcer, bleeding^d^	2/68 (2.9 %)	2.9 %
	64		20 Gy 1 fx^b^ + 50 Gy^c^ (median)	135	None	100 %	Duodenal ulcer, fatal hemorrhagic shock, grade 5	1/64 (1.6 %)	1.6 %
Goldson et al. [22]	19	Unknown	22.5 Gy 1 fx^b^ (median)	102	None	None	GI ulcers, bile duct obstruction^d^	2/19 (10.5 %)	10.5 %

^a^SBRT

^b^IORT

^c^EBRT

^d^Grade not specified, but presumed to be a grade ≥ 3 toxicity

**Table 2:** Canine GI toxicity rates from studies using hypofractionated radiotherapy

Author (reference)	N^c^	Median follow-Up (months)	Radiotherapy	*EQD*_4_^1.8^	Chemo (%)	Surgery (%)	Late GI complications	Complication rate	Total GI toxicity rate
Ahmadu-Suka et al. [23]	4	4.8	17.5 Gy 1 fx^a^ + 50 Gy^b^ (exact)	126	none	100 %		0/4 (0 %)	0 %
	3		25 Gy 1 fx^a^ + 50 Gy^b^ (exact)	177	none	100 %		0/3 (0 %	0 %
	4		32.5 Gy 1 fx^a^ + 50 Gy^b^ (exact)	256	none	100 %	Non-perforated duodenal ulcers^d^	3/4 (75 %)	100 %
							Perforated ulcers^d^	1/4 (25 %)	
	4		40 Gy 1 fx^a^ + 50 Gy^b^ (exact)	355	none	100 %	Non-perforated duodenal ulcers^d^	1/4 (25 %)	100 %
							Perforated ulcers^d^	3/4 (75 %)	
Halberg et al. [24]	2	6	30 Gy 1 fx^a^ (exact)	176	none	100 %	Duodenal ulceration, grade 3	2/2 (100 %)	100 %

^a^IORT

^b^EBRT

^c^Number of dogs surviving ≥ 3 months

^d^Grade not specified

### Toxicity

Serious gastrointestinal toxicities consisted of ulcers, hemorrhages, obstructions, strictures, and perforations. The median late grade 3 or greater GI toxicity among all studies was 7.4 % (range: 0–32.7 %). The most frequently reported toxicity, with a median rate of 9.1 % (range: 3.9–20 %), was ulcers that were neither hemorrhagic nor perforated. The highest rates of ulceration occurred in the two cohorts of Nishimura and colleagues (9.9 % and 20 %) [20]. Not surprisingly, the median dose used in these two cohorts was higher than any other study that reported non-hemorrhagic and non-perforated ulcers (EQD_4_^1.8^ 178 and 209, respectively). The patients in the cohort that experienced a 20 % ulcer rate had resectable tumors and underwent either pancreatectomy or pancreaticoduodenectomy, while the cohort with a 9.9 % ulceration rate had unresectable tumors [20]. Hemorrhages, which consisted of hemorrhagic ulcers or erosions in the stomach, duodenum, or colon, occurred at a median of rate of 4.6 % (range: 0.95–11.9 %). A median of 3.6 % (range: 1.3–5.0 %) and 3.1 % (range: 0.60–5.2 %) of patients developed perforations and strictures/obstructions, respectively.

There were four reported fatalities in the studies analyzed. Willett and colleagues reported two deaths from treatment-related upper GI bleeding at 37 and 53 months after treatment [18]. The patients in this study all received IORT, EBRT, and 5-fluorouracil with a median EQD_4_^1.8^ of 133 Gy. Okamoto and colleagues reported a fatality 11 months after treatment from a hemorrhagic ulcer in a patient that had undergone a distal pancreatectomy previously [21]. The exact dose used in this patient was not reported, but the median dose of the cohort was 20 Gy IORT and 50 Gy EBRT postoperatively (total EQD_4_^1.8^ 135 Gy). This study also reported a fatality due to an esophageal variceal rupture 38 months after treatment. This patient also underwent pancreaticoduodenectomy. The cause of death was deemed to be related to irradiation as the patient was found to have a portal vein thrombosis and obstruction on CT scan.

### Canine data

Toxicity rates from canine studies are summarized in Table 2. Ahmadu-Suka and colleagues treated the abdomen of dogs with a single fraction IORT dose up to 40 Gy followed by 50 Gy of EBRT two weeks after surgery [23]. Dogs treated with 17.5 or 25 Gy IORT disease (EQD_4_^1.8^ 126 and 177, respectively) showed mucosal atrophy, but did not show any duodenal ulcers on autopsy 4.5 months after treatment. Dogs surviving greater than 3 months and treated with 32.5 Gy IORT (EQD_4_^1.8^ 256) all showed ulcers on autopsy, and 25 % were perforated. Among the dogs surviving longer than 3 months and treated with 40 Gy IORT (EQD_4_^1.8^ 355), all experienced duodenal ulcers, and 75 % were perforated.

Halberg and colleagues examined the duodenum of dogs 6 months after treatment with 30 Gy IORT (EQD_4_^1.8^ 176) in the presence of intraluminal WR-2721 or vehicle control [24]. The two dogs in the control cohort that survived to 6 months both showed grade 3 duodenal ulcers on autopsy.

### NTCP model

**Fig. 1:**
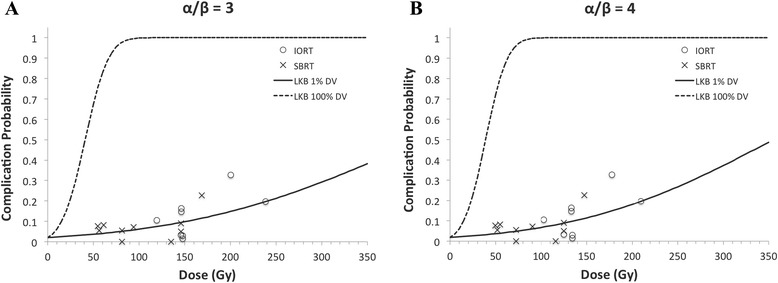
Human late GI toxicity as a function of fraction corrected median or mean study dose (*EQD* ^1.8^) for α/β of 3 (**a**) and 4 (**b**) and corresponding LKB model assuming duodenal volume receiving the prescription dose of 1 % and 100 %. DV = duodenal volume

Lyman NTCP model parameters and corresponding 95 % confidence intervals for human GI toxicity data are summarized in Table 3. Because dose volume histograms were not available, we estimated parameters assuming 1 to 5 % of the duodenum received the study’s prescribed dose. If multiple doses or fractionation schemes were employed in a single study, the median study dose was used for our analysis. In some instances, the median study dose was not provided and could not be calculated because the exact dose distribution was not reported. In these limited cases, the mean dose as reported by the authors was used. According to published reports, an α/β ratio of 3.0 or 4.0 is appropriate for bowel toxicity, thus we estimated the median or mean EQD_4_^1.8^ using a α/β ratio of 3 or 4 (Table 3) [25, 26]. Figure 1 shows overall GI toxicity rate as a function of median or mean dose for each study and the maximum likelihood fit of the LKB model for α/β ratio of 3 and 4 and duodenal volume 1 % and 100 % (Fig. 1).

**Table 3:** NTCP LKB parameters of pooled human GI toxicity studies. An α/β ratio of 3 or 4, median or mean study EQD, and duodenal volumes of 1–5 % were used for analysis. Parameters and corresponding 95 % confidence intervals as calculated by the profile likelihood method are shown

NTCP model assumptions		NTCP model parameters		
α/β	Volume	n (95 % CI)	m (95 % CI)	TD50 (95 % CI)
3	1 %	0.50 (0.47–0.53)	0.49 (0.46–0.52)	41.0 (36.2–47.4)
3	2 %	0.38 (0.35–0.42)	0.49 (0.46–0.52)	91.0 (80.3–105.4)
3	3 %	0.42 (0.38–0.46)	0.49 (0.46–0.52)	95.0 (83.9–110.1)
3	4 %	0.50 (0.46–0.54)	0.49 (0.46–0.52)	81.9 (72.3–94.9)
3	5 %	0.63 (0.58–0.67)	0.49 (0.46–0.52)	63.0 (55.6–72.9)
4	1 %	0.48 (0.45–0.51)	0.48 (0.45–0.51)	39.0 (34.7–44.7)
4	2 %	0.59 (0.56–0.62)	0.48 (0.45–0.51)	35.0 (31.1–40.1)
4	3 %	0.45 (0.42–0.50)	0.48 (0.45–0.51)	71.0 (63.1–81.5)
4	4 %	0.45 (0.42–0.50)	0.48 (0.45–0.51)	81.0 (71.9–92.9)
4	5 %	0.43 (0.40–.48)	0.48 (0.45–0.51)	95.0 (84.4–109.1)

### Univariate logistic regression

We performed univariate analysis to determine the effect of follow-up time, dose, overall survival, primary modality of radiotherapy (SBRT vs. IORT), number of fractions, percentage of patients receiving chemotherapy, and percentage of patients undergoing surgery on the overall gastrointestinal toxicity rate. Dose was the only significant predictor of GI toxicity (Table 4).

**Table 4:** Univariate analysis. The impact of several study parameters on toxicity was analyzed using univariate analysis

Variable	*P* value
Median follow-up	0.82
Median radiation dose	**0.01**
Number of dose fractions	0.53
Modality (IORT vs SBRT)	0.29
% of patients receiving chemotherapy	0.22
% of patients receiving surgery	0.98
Median overall survival	0.53

## Discussion

Tissue complications from radiation therapy can present in early and late phases. Early complications in the bowel are related to acute mucosal injury. Denudation of the rapidly dividing epithelial cells that line the gastrointestinal tract can result in nausea, vomiting, gastritis, and/or diarrhea. These early effects are usually transient, beginning less than a week after the first dose and resolving soon after the last treatment. Late complications, on the other hand, may appear within a few months of treatment and result from fibrotic changes to the bowel and its vasculature. Ischemia and fibrosis lead to mucosal atrophy, ulceration, tissue breakdown, perforation, inflammation, and the formation of strictures, obstructions, and adhesions. Perforation of the duodenum is particularly dangerous due to its close proximity to the vessels of the mesentery and their branches. Better characterization of late gastrointestinal toxicity is important for safe treatment of patients with pancreatic cancer.

In an attempt to characterize late GI toxicity in hypofractionated treatment of pancreatic cancer, we compiled published gastrointestinal late toxicity data from multiple institutions. We reasoned that this approach, which in total encompasses over a thousand patients across multiple studies and institutions, would reveal gastrointestinal toxicity trends and patterns that may not be apparent in studies with smaller cohorts. For example, while ulcers were the most common observed toxicities at a rate of 9.9 %, hemorrhages, perforations, and obstructions occurred at a rate of 3–5 % and might not be appreciated in smaller studies.

We also examined the relationship between radiation dose and late gastrointestinal complications. We specifically examined studies that used hypofractionated treatment irrespective of modality (between 1 and 5 fractions). Several patients also received conventionally fractionated radiotherapy. We calculated the EQD_4_^1.8^ for all radiation treatments given to the volume, and summed them arithmetically. Whether the linear-quadratic model accurately represents the true biologic efficacy of hypofractionated radiation treatments is an issue of debate, especially when the fraction size is large [27]. In our analysis, we noticed a clear relationship between dose and toxicity (Fig. 1). Univariate analysis of multiple study characteristics identified dose as a significant predictor of toxicity (Table 4). A similar literature analysis attempting to identify a therapeutic window for SBRT use in pancreatic adenocarcinoma by Brunner and colleagues also identified a significant relationship between dose and gastrointestinal toxicity [28]. The authors compiled gastrointestinal toxicity from 16 literature reports following SBRT of the pancreas. Linear regression showed a positive correlation between grade ≥ 3 toxicity and study EQD_3_^2^ (R^2^ = .77). A 5 % rate of grade ≥ 3 toxicity was associated with an EQD_3_^2^ of 80 Gy. Interestingly, examining the raw data for Fig. 1 based on a similar EQD_3_^1.8^, the 5 % complication rate is reached at 80 Gy, and with an EQD_4_^1.8^ the 5 % complication rate is reached at 75 Gy. Therefore, both studies seem consistent in this respect.

Because dose volume histograms were not available, we estimated NTCP model parameters assuming duodenal volumes of 1–5 % received the prescription dose, which is consistent with clinical practice and as reported in some of the series we examined. For example, Chang and colleagues as well as Schellenberg and colleagues treated patients with 25 Gy in a single fraction using SBRT. In both studies, the volume of duodenum receiving more than 22.5 Gy was less than 5 % [10, 13]. Willett and colleagues as well as Goldson and colleagues retracted normal GI structures away from the cylinder applicator when treating patients with IORT [18]. Nishimura and colleagues similarly retracted normal GI structures outside the treatment field. In cases where the GI structures could not be completely excluded, a smaller dose was applied to the entire field and a higher dose applied to the central region targeting the tumor (field-in-field). Thus, an upper limit of 5 % of duodenal volume exposed to the maximum dose seems reasonable.

Derived NTCP LKB model parameters for the compiled toxicity data set are shown in Table 3. TD_50_ values ranged from 35 to 95 Gy and represent clinically plausible constraints. Early work by Burman *et al.* analyzing toxicity data compiled by Emami and colleagues obtained TD_50_ values of 55 for small intestine and 65 Gy for stomach [29, 30]. Prior and colleagues attempted a similar analysis on compiled duodenal and small bowel toxicity using a modified linear quadratic model of multiple fractionation schedules and derived a TD_50_ value of 60.9 Gy [31]. Murphy and colleagues derived NTCP parameters for patients receiving a single 25 Gy dose using SBRT for pancreatic cancer and obtained a TD_50_ value of 24.6 Gy (single fraction) [32]. Not surprisingly, our TD_50_ values were highly dependent on duodenal volume assumptions (Table 3). Indeed, Murphy and colleagues performed a dose volume analysis of GI toxicity in patients treated with 25 Gy in a single fraction and found a significant association between duodenal volume and toxicity [32]. Availability of DVHs corresponding to toxicity would allow the prediction of more accurate and clinically applicable NTCP parameters and highlights the need for multi-institutional treatment toxicity databases. While differences in treatment (i.e., use of chemotherapy or surgery) or patient characteristics across institutions may affect toxicity rates, our analysis suggests that dose is the primary predictor of toxicity (Table 4). Other important and potentially confounding variables such as median follow-up, median survival, use of chemotherapy, or surgery, were not significant.

There are many limitations to our approach. We examined the relationship of total dose to late GI toxicity rate and did not study the relationship of dose to the specific type of complication reported (e.g., ulceration, bleeding, perforation). Many of the studies we analyzed reported a range of doses given to their patient cohort, and the majority did not report the specific dose corresponding to each reported toxicity. Thus, identifying threshold doses for serious GI complications was difficult. To circumvent this limitation in reporting, median study dose was used. This approach clearly has the potential to confound the relationship between radiation dose and toxicity rate. Our analysis is also limited by the lack of consideration of the variability in the partial volume of duodenum irradiated among different studies. While we attempted to model the effect of partial volume by assuming uniform volumes of 1–5 % across all studies, assuming a single volume for all studies will likely introduce error. Differences among reporting strategies, modality, contouring of normal structures, and general patient variability could all be sources of variability within the data. We attempted to identify sources of variability within the data by performing univariate regression analysis, which identified fraction-adjusted dose as the only significant predictor of toxicity.

## Conclusions

In conclusion, we have aggregated and analyzed late gastrointestinal toxicity data from studies utilizing hypofractionated treatment for pancreatic cancer. Reported toxicities consisted of ulcers, hemorrhages, obstructions, strictures, and perforations. Non-hemorrhagic ulcers were the most frequent complication and occurred in approximately 9.1 % of patients. We also observed a relationship between study dose and rates of late grade ≥ 3 gastrointestinal toxicity and have derived preliminary NTCP LKB model parameters. Additional studies using individual patient data, with access to dose volume distributions and individual histories and toxicity reports, would allow us to better characterize the dose–response relationship for gastrointestinal toxicity in hypofractionated radiation treatment. A multi-institutional prospectively maintained treatment database would allow a more accurate analysis to be performed in the future.
